# Data of the effects of acetone fraction from *Sechium edule* (Jacq.) S.w. edible roots in the kidney of endothelial dysfunction induced mice

**DOI:** 10.1016/j.dib.2018.03.051

**Published:** 2018-03-16

**Authors:** Celeste Trejo-Moreno, Gabriela Castro-Martínez, Marisol Méndez-Martínez, Jesús Enrique Jiménez-Ferrer, José Pedraza-Chaverri, Gerardo Arrellín, Alejandro Zamilpa-Álvarez, Omar Noel Medina-Campos, Galia Lombardo-Earl, Gerardo Joel Barrita-Cruz, Beatriz Hernández, Christian Carlos Ramírez, María Angélica Santana, Gladis Fragoso, Gabriela Rosas

**Affiliations:** a Facultad de Medicina, Universidad Autónoma del Estado de Morelos, Cuernavaca, Morelos CP 62350, Mexico; b Laboratorio de Farmacología, Centro de Investigaciones Biomédicas del Sur, Instituto Mexicano del Seguro Social, Xochitepec, Morelos CP 62790, Mexico; c Facultad de Química, Universidad Autónoma de México, Coyoacán, Ciudad de México CP 04510, Mexico; d Facultad de Ciencias de la Salud, Universidad Panamericana, Ciudad de México CP 03920, Mexico; e Facultad de Medicina, Universidad Nacional Autónoma de México, Coyoacán, Ciudad de México CP 04510, Mexico; f Centro de Investigación en Dinámica Celular, Universidad Autónoma del Estado de Morelos, Av. Universidad 1001, Chamilpa, Cuernavaca, Morelos CP 62209, Mexico; g Instituto de Investigaciones Biomédicas, Universidad Nacional Autónoma de México, Coyoacán, Ciudad de México CP 04510, Mexico

## Abstract

Endothelial dysfunction induced by Angiotensin II (AG II) plays an important role in the pathogenesis of hypertension and is accompanied by a prooxidative condition, which in turn induces an inflammatory state, vascular remodeling, and tissue damage including the kidney (Schmitt and Dirsch, 2009) [1]. New drugs that can control several of these pathologies are required. *Sechium edule* has been reported to possess antioxidant, anti-inflammatory and antihypertensive activity (Ibarra-Alvarado et al., 2010) [2]. This paper contains data complementary to those published in Journal of Ethnopharmacology (Moreno et al., 2018) [3], evaluating the effect in kidney of hypertensive mice of the acetone fraction from *S. edule* to control de pro-oxidative state, reduction of the inflammatory adhesion molecule (ICAM) and recruitment of inflammatory cells.

**Specifications Table**

**Table:** 

Subject area	Medicine
More specific subject area	Use of extracts of *Sechium edule* to control endothelial dysfunction
Type of data	Text file and figures
How data was acquired	The Activity of antioxidant enzymes was measured by enzymatic reactions measuring the end product using a Spectrophotometer and analyzed in Excel and Microphotographs were acquired with a e Nikon ECLIPSE 80i microscope. The images were analyzed with the Metamorph software, v. 6.1.survey.
Data format	Analyzed
Experimental factors	The acetonic fraction of *Sechium edule* roots (rSe-ACE) were obtained and tested in its anti-oxidant, anti-inflammatory and anti-hypertensive capacity in a murine model of endothelial dysfunction [3].
Experimental features	Hypertension was induced in female mice by the chronic administration of angiotensin II for 10 weeks [1]. Treatments (Losartan or the acetonic fraction of *Sechium edule*) were coadministrated also for the 10 weeks. At the end of the experiment, kidneys were obtained and processed for biochemical or microscopic studies
Data source location	*Sechium edule* (Jacq.) Sw. roots were collected in the community of Tuxpanguillo, Veracruz, Mexico (18°47′00.5″N, 97°00′17.5″W, at 1721 m above mean sea level) in a period from April through May.
Data accessibility	Data is with this article.

**Value of the data**

• A model of endothelial dysfunction is induced in rats by the chronic administration of angiotensin II.
• rSe-ACE is able to control Endothelial dysfunction (ED) and its associated pathologies.
• rSe-ACE is a good candidate for the development of a phytochemical medicine for ED.

## 1 Data

**Fig. 1:**
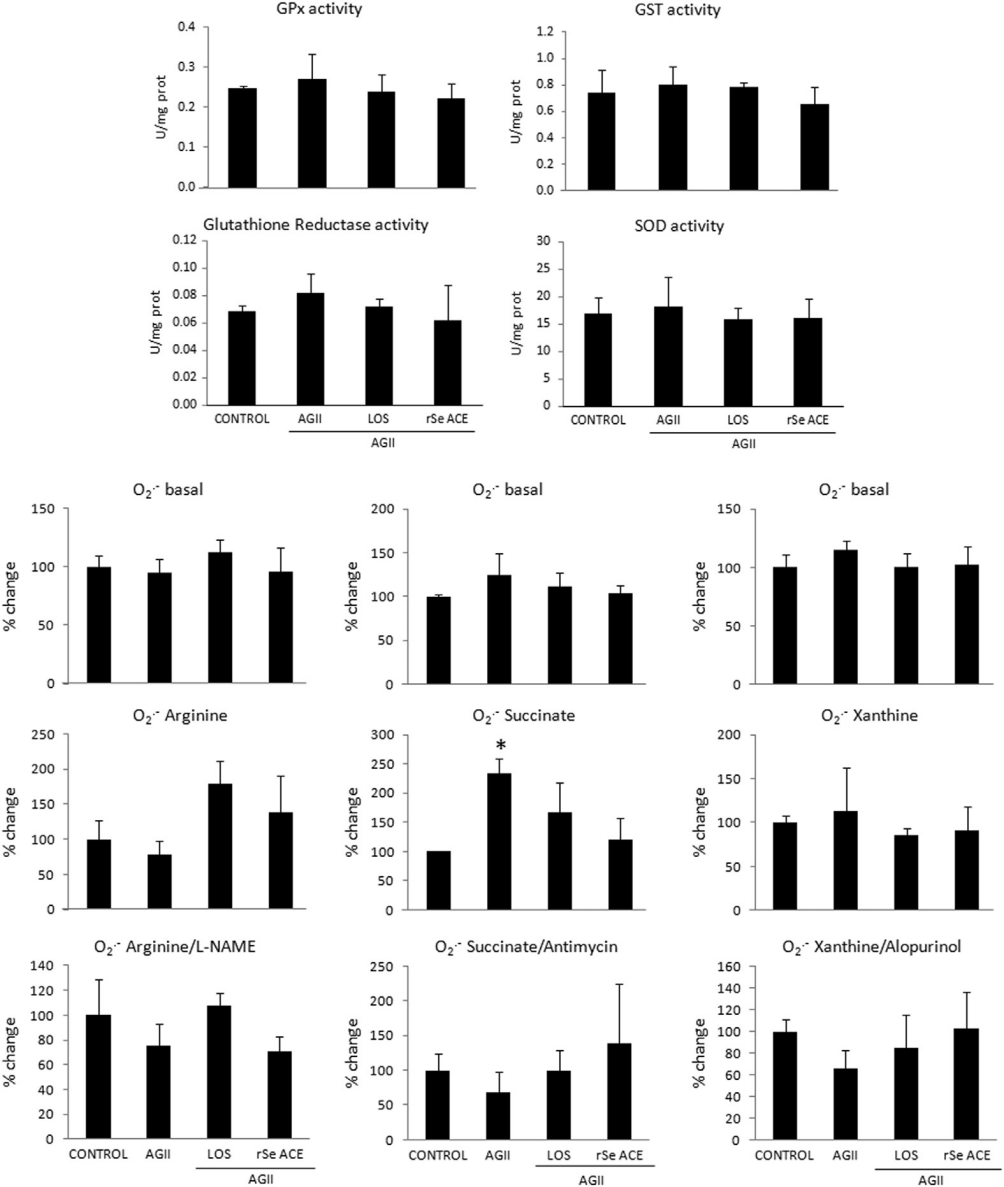
**Effect of rSe ACE on renal activity of antioxidant enzymes in mice.** Data are expressed as mean±SD and were analyzed by ANOVA and a post-hoc Tukey-Kramer test. *Statistically significant differences with respect to control (*P* < 0.05).

**Fig. 2:**
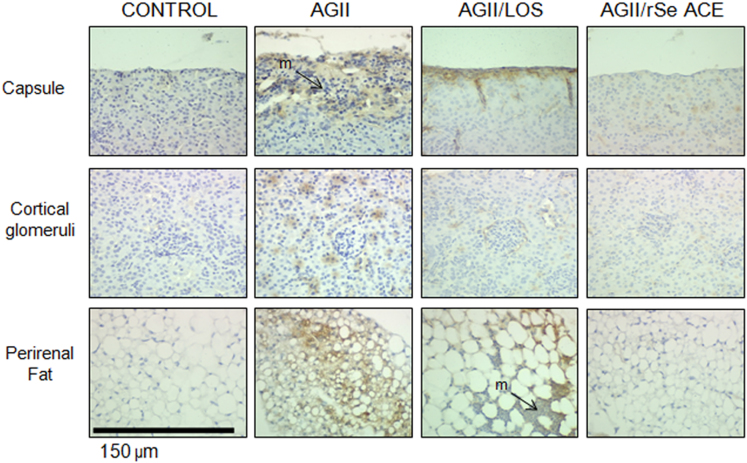
**ICAM-1 expression by immunohistochemistry.** ICAM-1 expression in Renal capsule, Cortical glomerulus and Perirenal fat tissue of control intact mice and of AgII treated mice with or without Losartan (LOS) or rSeACE. 40× microphotographs. m: mononuclear cells.

**Fig. 3:**
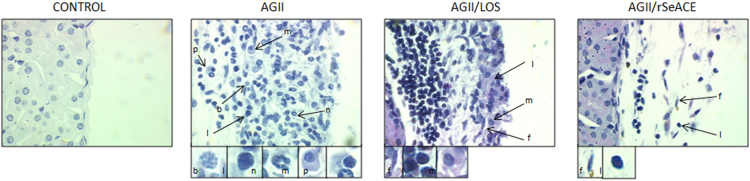
**Cell populations in the renal capsule**. Cellular infiltrate in the renal capsule from the control mice and from the AGII-treated group further treated with or without Losartan or rSeACE. B: basophile; F: fibroblast; L: lymphocyte; N: neutrophil; Macrophage; P: plasma cell. Micrographs were taken at 100X.

The dataset of this article provides information on the effects of the acetonic fraction of *Sechium edule* roots in the activity of antioxidant enzymes (Fig. 1), in the expression of the inflammatory adhesion molecule ICAM 1 (Fig. 2) and in the recruitment of inflammatory cells on renal capsule (Fig. 3) in kidney of endothelial dysfunction induced mice through the administration of Angiotensin [1].

## 2 Material and methods

### 2.1 Animals and experimental groups

Three groups of hypertensive female C57BL/6J mice, five mouse each, were coadministered with losartan (10 mg/kg) diluted in carboxymethylcellulose (CMC) 0.2% [4] or rSe-ACE (10 mg/kg) [2] diluted in water orally for 10 weeks. A fourth group was treated only with saline solution. Mice were regarded as hypertensive when blood pressure increased by 15% or more with respect to baseline blood pressure (systolic or diastolic). Mice were killed by CO_2_ inhalation and exsanguinated at the end of the experiment

### 2.2 Tissue preparation for biochemical analysis

Kidneys were homogenized in ice-cold HEPES buffer (HEPES 25 mM, EDTA 1 mM, and phenylmethylsulfonyl fluoride [PMSF] 0.1 mM). Homogenates were centrifuged at 6000×*g* for 5 min at 4 °C and supernatants were recovered. Protein concentration was determined by the Lowry method.

#### 2.2.1 Measurement of O_2_^−^ production

Dihydroethidium oxidation (DHE) to ethidium (Eth) was used to measure O_2_^−^ production. Supernatants (10 µl) were incubated with DHE (0.02 mM), salmon testis DNA (0.5 mg/mL), and the corresponding substrate for xanthine oxidase (XO), mitochondrial respiratory enzymes, or nitric oxide synthase (NOS). Eth-DNA fluorescence was measured at an excitation wavelength of 480 nm and an emission wavelength of 610 nm at 37 °C for 30 min using a multimode microplate reader (Synergy HT, Biotek, Winooski, VT). Xanthine (0.1 mM) was used as a substrate for XO, and allopurinol (0.2 mM) was used as an inhibitor. Succinate (5 mM) was used as a substrate for mitochondrial O_2_^−^ production, and antimycin (0.05 mM) was used to block the respiratory chain. L-arginine (L-Arg, 1 mM) was used as a substrate for NOS, while NG-nitro-L-arginine methyl ester (L-NAME, 1 mM) was used to block NOS activity. A blank without sample was run to subtract background fluorescence from each sample reading. Enzyme activity is expressed with respect to control readings.

#### 2.2.2 Glutathione peroxidase assay

The activity of renal glutathione peroxidase (GPx) was assayed by measuring NADPH disappearance by optical density (OD) reading at 340 nm. GPx uses GSH to reduce tert-butyl hydroperoxide, thereby producing GSSG, which is readily reduced to GSH by GR using NADPH as a reducing equivalent donor. Blank reactions with the sample replaced by distilled water were subtracted from each assay reading. Data were expressed as units per mg protein.

#### 2.2.3 Glutathione reductase assay

The activity of glutathione reductase (GR) was assayed using oxidized glutathione as a substrate. Briefly, GR reduces GSSG to GSH at the expense of NADPH; the disappearance of NADPH can be detected by reading OD at 340 nm.

#### 2.2.4 Glutathione-S-transferase assay

The activity of glutathione-S-transferase (GST) was evaluated using a reaction mixture consisting of potassium phosphate buffer 50 mM (pH 6.5), reduced glutathione 2 mM, and 1-chloro-2,4-dinitrobenzene (CDNB) 1 mM. Supernatants (0.02 ml) were added to 0.98 ml of the reaction mixture, and absorbance was recorded at 340 nm for 3 min. Enzymatic activity was calculated as µmol CDNB conjugate formed/min/mg protein using a molar absorptivity of 9.6 × 10^3^/M/cm.

#### 2.2.5 Superoxide dismutase assay

The total activity of Superoxide Dismutase (SOD) was evaluated employing a competitive inhibition assay using a xanthine-XO system to reduce nitroblue tetrazolium (NBT). The percentage of NBT reduction in the sample-free control tube was 100%. The amount of protein that inhibited NBT reduction by 50% of the maximum reading was defined as one unit of SOD activity. Results were expressed as U/mg protein.

## 3 Histopathology and immunohistochemistry

After the last blood pressure measurement, mice were anesthetized with sodium pentobarbital (30 mg/kg i.p.) and perfused with ice-cold PBS (NaCl 140 mM, KCl 2 mM, and K_2_HPO_4_ 1.15 mM). Kidneys were removed. The organs were fixed in Zamboni solution (formaldehyde 2.0%, picric acid 0.2%, pH 7.0). Tissues were then dehydrated and embedded in paraffin. Tissue sections (5 μm) were transferred to poly-L-lysine-coated slides (Sigma) before being deparaffinized and rehydrated. For histopathological studies, the slides were stained with PAS stain. For Immunohistochemistry, kidney slides were deparaffined, rehydrated, and incubated with H_2_O_2_ 3%, albumin 5%, and Tween 20-PBS 1%. After treatment, tissue sections were incubated overnight with rat anti-mouse ICAM-1 (eBioscence) diluted 1:100 in albumin 0.1% and Tween 20-PBS 0.05%. After wash with PBS, tissues were incubated with 50 µl of biotinylated goat anti-rat IgG (MP Biomedicals) antibody, followed by HRP-labeled streptavidin solution (MP Biomedicals) at 37 °C for 30 min, and developed with 3,3 diaminobenzidine (ZYMED, San Francisco, CA). The slides were counterstained with hematoxylin and photographed using a Nikon ECLIPSE 80i microscope. The images were analyzed with the Metamorph software, v. 6.1.
